# Real-Time Curvature Detection of a Flexible Needle with a Bevel Tip

**DOI:** 10.3390/s18072057

**Published:** 2018-06-27

**Authors:** Bo Zhang, Fangxin Chen, Miao Yang, Linxiang Huang, Zhijiang Du, Lining Sun, Wei Dong

**Affiliations:** State Key Laboratory of Robotics and System, Harbin Institute of Technology, Harbin 150001, China; dongway@gmail.com (B.Z.); chenfx@hit.edu.cn (F.C.); yangmiaohit@hit.edu.cn (M.Y.); h_linxiang@163.com (L.H.); duzj01@hit.edu.cn (Z.D.); lnsun@hit.edu.cn (L.S.)

**Keywords:** bevel-tip flexible needle, puncture, curvature detection

## Abstract

As one of the major methods for the diagnosis and treatment of cancers in their early stages, the percutaneous puncture technique has bright prospect in biopsy, ablation, proximity radiotherapy, and drug delivery. Recent years, researchers found the flexible needle cannot realize feedback control during the puncture surgeries only by path planning. To solve this problem, the flexible needle is tried to achieve real-time detection in this paper. Compared with previous methods, the strain gauges glued on the needle surface rather than the medical imaging techniques is used to collect the information to reconstruct the needle curve, which is benefit to integrate the whole system and obtain a more simple and accurate closed-loop control. This paper presented the math model of curve fitting and analyzed the causes of curve fitting errors. To verify the feasibility of this method, an experiment setup was built. Results from the experiments validated the solution in this paper to be effective.

## 1. Introduction

The percutaneous puncture technique, a typical minimally invasive surgery, makes smaller wounds, causes less postoperative complications, and is easier for patients to recover compared with traditional surgeries. It uses a needle to penetrate into the skin to detect and treat diseases [1,2,3,4]. The percutaneous puncture technique plays a positive role in biopsy, ablation, proximity radiotherapy, and drug delivery in clinical applications. It has tremendous advantages especially in the diagnosis and treatment of cancers in their early stages [5].

The needle used in today’s operating room are rigid. Since it can only move straight, the rigid needle can be only utilized in some simple operations. For some complicated cases where human tissue might block their way, it is useless. As a result, researchers come up with the idea of a bevel-tip flexible needle, whose end has an asymmetric bevel, making it curves as it goes ahead in the skin, so it can avoid the tissue and arrive at the target place successfully [6,7,8,9,10].

Scientists have put great efforts in the modeling of a needle’s force-position relations, hoping to achieve accuracy path planning of the needle through a high precision math model. Simon P. DiMaio and S. E. Salcudean [11] first proposed the idea of “control of flexible needles” in their study of rigid needles. Daniel [12] analyzed the forces and deflection of the flexible needle in the tissues, built the virtual spring model of the needle, and studied its path planning and control strategies. Robert J. Webster [13] proposed an incomplete unicycle model and a bicycle model according to the Lie group theory and obtained the model parameters from experiments. They concluded the effects of puncture speed and bevel-tip angle on a needle’s trajectory. Based on the bicycle model, Engh [14] studied the path of the flexible needle under different motor duty ratios and tried to use various arcs to avoid obstacles. Riviere [15] proved that the bevel-tip flexible needle can be used for obstacle avoidance during puncture operations based on trajectory planning.

However, no matter how accurate the path planning is, the safety cannot be guaranteed in puncture. The path controlling based on the real-time detection is in great request. Integrating medical imaging techniques, such as Ultrasonic image, CT, and MRI, into control strategies is a main technological means for feedback of the needle positions. Ultrasound image is convenient and has good real-time response, no radiation, and no damage to the human body, but it is less satisfactory in accurate positioning and penetration [16]. CT has radiation damages to patients and is usually used in the imaging of bones [17]. MRI has a high cost and requires the puncture system to be made up of non-ferromagnetic materials, which limits its application [18]. Above all, it is hard to get a stable and accurate needle image as a result of the needle’s long and thin structure applying the medical imaging techniques [19]. Therefore, combining medical imaging equipment to recognize and track the path of the needle is not a satisfactory solution.

A better thought for the real-time needle curve feedback is to integrate the sensors into the flexible needle. Park [20] embedded optical fiber sensor inside a rigid needle to detect its shift. Although the system was complicated, their work provided a new method for the detection of a needle. The key challenge integrating the sensor and the needle is to find appropriate sensing units which can be arranged in the needle whose geometric dimension is extremely limited. It is also fundamental to optimize the layout of the sensors on the needle to meet the requirements of low cost and high precision at the same time. This paper tried to use resistance strain gauges to solve the problem mentioned above.

The rest of the paper is arranged as follows: Section 2 introduces the bevel-tip flexible needle detection system based on resistance strain gauges. Section 3 is the modeling of the detection method. The experiments and results are presented in Section 4. Finally, Section 5 gives the conclusion of this paper.

## 2. System Design

**Figure 1:**
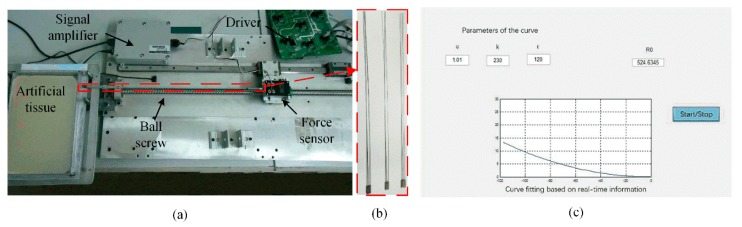
The experiment setup. (**a**) The hardware configuration; (**b**) The flexible needles used; and (**c**) The GUI.

The bevel-tip flexible needle detection system consists of the hardware and the software. The hardware includes a bevel-tip flexible needle, an actuation device, a data acquisition card (NI PCI-6251), a 24V DC power supply, and artificial human tissue. The software includes the DAQ model from LabVIEW and MATLAB-GUI, as show in Figure 1c. In implementation, the main control computer sends control commands to the motor driver via the data acquisition card. The motor actuates the needle insertion via a lead screw. In some research cases, the insertion force information is required (not included in this paper), therefore, a force sensor and a signal amplifier are also involved in the experiment setup.

### 2.1. Fabrication of the Needle

**Figure 2:**
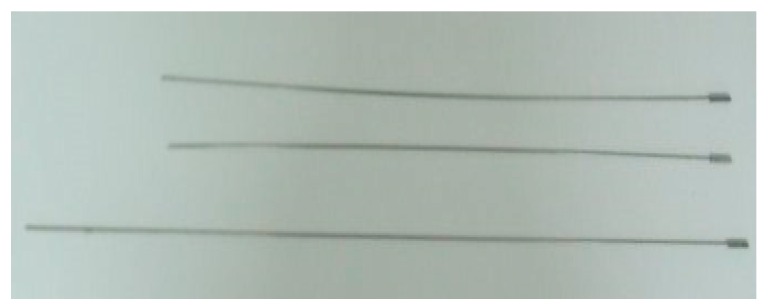
Flexible needles with a bevel tip.

The bevel-tip needle is made of nickel-titanium wire, hyper-elastic material, which is able to deflect in tissue. Figure 2 is the needles for puncture. The diameters of needles medical used vary from 0.2 mm to 4.5 mm. Considering experimental conditions, the diameter of the needle in this paper is 1.2 mm. The bending radius will increase with the angle of the bevel increase (i.e., less bended). The bevel-tip of the needle used in the experiment is 15°. Other parameters of the needle are listed in Table 1.

**Figure 3:**
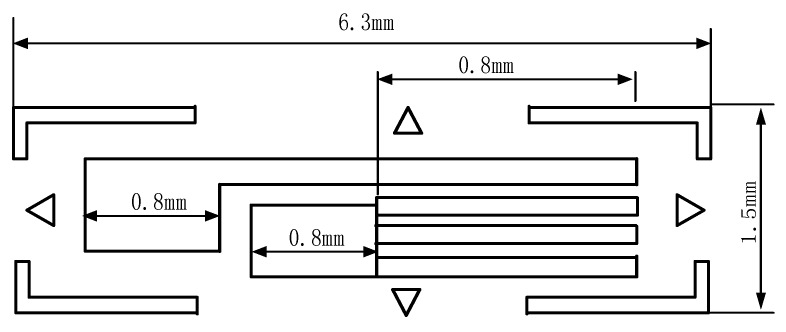
The size of the resistance strain gauge.

**Figure 4:**
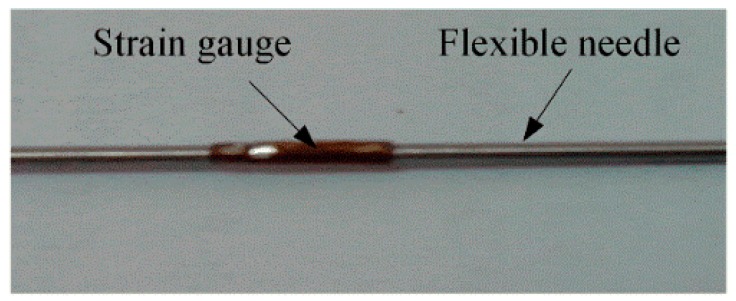
Needle with a strain gauge.

The size of resistance strain gauges is limited by the size of the needle. This paper uses the high precision strain gauge (ZF120-3AA-A (9)-G100) of Electrical Measurement, whose size is shown in Figure 3. The bridge circuit comprises three 120 Ω resistance. The gauge is glued at the discrete point of the needle, parallel to the axis. It is obvious that for a single curvature segment, one gauge is enough for the shape detection, where the discrete point is the center position along the axis direction of the needle. However, for a multi-curvature needle, more than one gauges is required to glue on the sensitive locations of different curvatures. For a specific insertion window and target location, the needle insertion trajectory can be planned in advance, (i.e., the situation of the curvatures can be known before implementing the needle insertion). Therefore, a customized configuration of the gauges distribution is feasible in practice. The needle with gauge is shown in Figure 4.

### 2.2. Artificial Tissue

Artificial tissue in experiments is used as the substitute for real human tissue, so it is important to make the artificial tissue have the same physical properties as real tissue.

#### 2.2.1. Fabrication of Artificial Tissue

**Figure 5:**
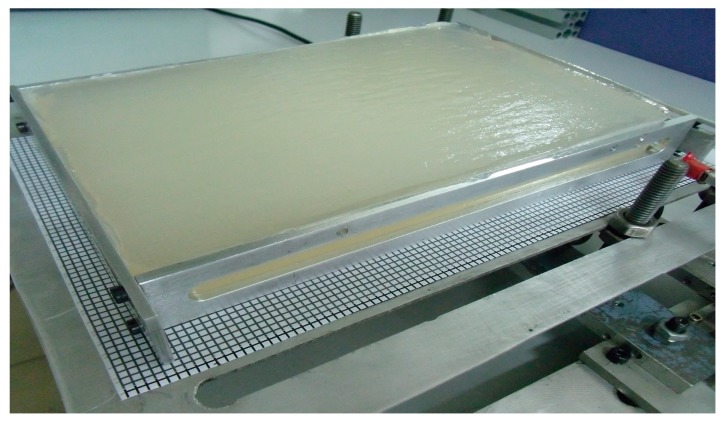
Artificial tissue.

The artificial human tissue was made of agar powder. Specifically, 15 g agar powder was dissolved in 1000 mL water, heated until boiling and lasted for 5 min, and then cooled down naturally, as shown in Figure 5.

#### 2.2.2. Measurement of Young’s Modulus of the Tissue

**Figure 6:**
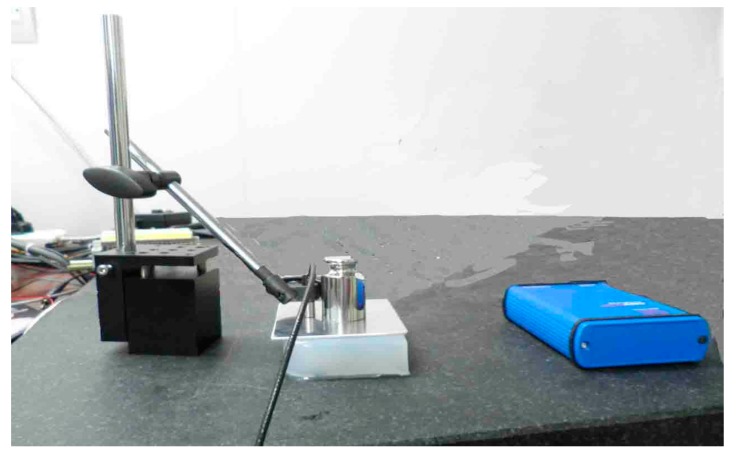
Measurement of Young’s modulus of the tissue.

The Young’s modulus of the tissue was measured by a capacitive sensor, as shown in Figure 6. Specifically, the deformation of the tissues were tested applying the capacitive sensor when the weights were added linearly on the upper surface of the tissue. The Young’s modulus of the tissue could be calculated with the weight and deformation. The Young’s modulus of human mammary tissue is about 7.5–66 kPa [21]. The Young’s modulus of the tissue tested in the experiment is 30 kPa.

## 3. Mathematic Model of Curve Fitting

When the needle deforms, the strain gauges generate voltages which are then transformed into curvature of the discrete points. The reconstruction of a needle on computer is performed based on the curvature and gauge positions by curve fitting. According to the shape of the needle, the curves are divided into plane curves and space curves.

### 3.1. Plane Curves Fitting

**Figure 7:**
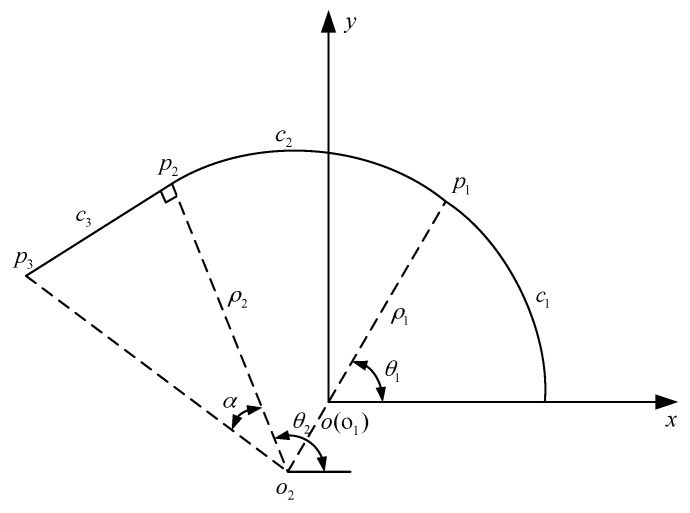
Schematic diagram of plane curve fitting.

Figure 7 is the schematic diagram of the plane curve fitting. The curvature is continuous because the needle is a smooth curve. Since the curvature of the needle doesn’t change too much in actual operation, the needle can be divided into some segments and it is assumed that each section has the same curvature. By doing so, the needle can be fitted by arcs. In Figure 7, *C_i_*, *P_i_* are the places where gauges are glued, and *O_i_* is the center of each arc. The center of the first arc is put at the origin of the coordinate system. *θ_i_* is the angle between x-axis and the line created by linking the end of an arc with its center. *ρ_i_* is the curvature radius of each arc.

For *C*_1_, the center is at the origin, so the function can be expressed as:

(1) $$\{\begin{array}{c} x=\rho_{1}\cos(\theta_{1})\\ y=\rho_{1}\sin(\theta_{1}) \end{array}$$

where 0 ≤ *θ* ≤ *θ*_1_. Then the coordinate of *P*_1_ is:

(2) $$\{\begin{array}{c} P_{1x}=\rho_{1}\cos(\theta_{1})\\ P_{1y}=\rho_{1}\sin(\theta_{1}) \end{array}$$

where *θ*_1_ = *C*_1_/*ρ*_1_. Each arc intersects one by one and is tangent to the next arc, so *O_i_*_+1_ must be on line *O_i_P_i_*. Then *P*_1_ in *C*_2_ can be written as:

(3) $$\{\begin{array}{c} P_{1x}=O_{2x}+\rho_{2}\cos(\theta_{1})\\ P_{1y}=O_{2y}+\rho_{2}\sin(\theta_{1}) \end{array}$$

Taking (2) into (3), the coordinate of *O*_2_ can be obtained:

(4) $$\{\begin{array}{c} O_{2x}=\rho_{1}\cos(\theta_{1})-\rho_{2}\cos(\theta_{1})\\ O_{2y}=\rho_{1}\sin(\theta_{1})-\rho_{2}\sin(\theta_{1}) \end{array}$$

When the center of *C*_2_ is known, the function in rectangular coordinate system can be written as:

(5) $$\{\begin{array}{c} x=\left(\rho_{1}-\rho_{2}\right)\cos(\theta_{1})+\rho_{2}\cos(\theta )\\ y=\left(\rho_{1}-\rho_{2}\right)\sin(\theta_{1})+\rho_{2}\sin(\theta ) \end{array}$$

where *θ*_2_ = *θ*_1_ + *C*_2_/*ρ*_2_. Therefore, the function of *C_i_* can be written as:

(6) $$\{\begin{array}{c} x=O_{(i-1)x}+\left(\rho_{i-1}-\rho_{i}\right)\cos(\theta_{i})+\rho_{i}\cos(\theta )\\ y=O_{(i-1)x}+\left(\rho_{i-1}-\rho_{i}\right)\sin(\theta_{1})+\rho_{2}\sin(\theta ) \end{array}$$

where *θ_i_* = *θ_i−_*_1_ + *C_i_*/*ρ_i_*(*i* ≥ 1). That is all about the fitting of arcs. 

For a straight line, for example segment 3 in Figure 7, the radius $\rho_{3}=\infty$. If the length of the line is known, then the end of the line *P*_3_ is:

(7) $$\{\begin{array}{c} P_{3x}=O_{2x}+O_{2}P_{3}\cos(\theta_{3})\\ P_{3y}=O_{2y}+O_{2}P_{3}\sin(\theta_{3}) \end{array}$$

where *O*_2_*P*_3_ = *ρ*_2_/sin(arctan(*C*_3_/*ρ*_2_)). Since the coordinate of the line’s origin (*P*_2*x*_, *P*_2*y*_) is already known, the function of the line can be easily obtained.

**Figure 8:**
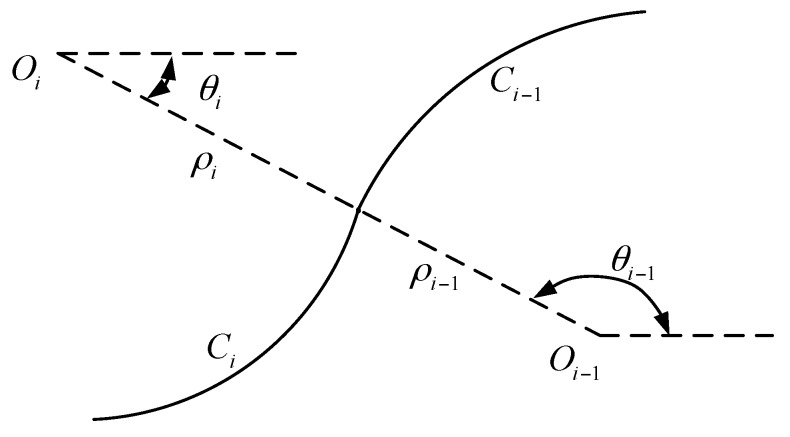
The situation when an anticlockwise arc is followed by a clockwise arc.

When an anticlockwise arc is followed by a clockwise arc, as shown in Figure 8, the function of clockwise *C*_i_ can be written if its center’s coordinate and the angle are known, which can be deduced by the follow equations:

(8) $$\{\begin{array}{c} O_{ix}=O_{(i-1)x}+\left(\rho_{i-1}+\rho_{i}\right)\cos(\theta_{i-1})\\ O_{iy}=O_{(i-1)y}+\left(\rho_{i-1}+\rho_{i}\right)\sin(\theta_{i-1}) \end{array}$$

It is easy to know that *θ_i_* = π − *θ_i−_*_1_*.*

### 3.2. Spatial Curves Fitting

The fitting of space curves has a similar process with plane curves. For space curves, the arcs can be in any plane in space. Under this circumstance, two vertical planes are chosen. Project the arc onto the two planes and solve the curvature of the arcs on both planes. Then calculate the vector sum of the two curvature values, which will be the curvature of the space curve.

**Figure 9:**
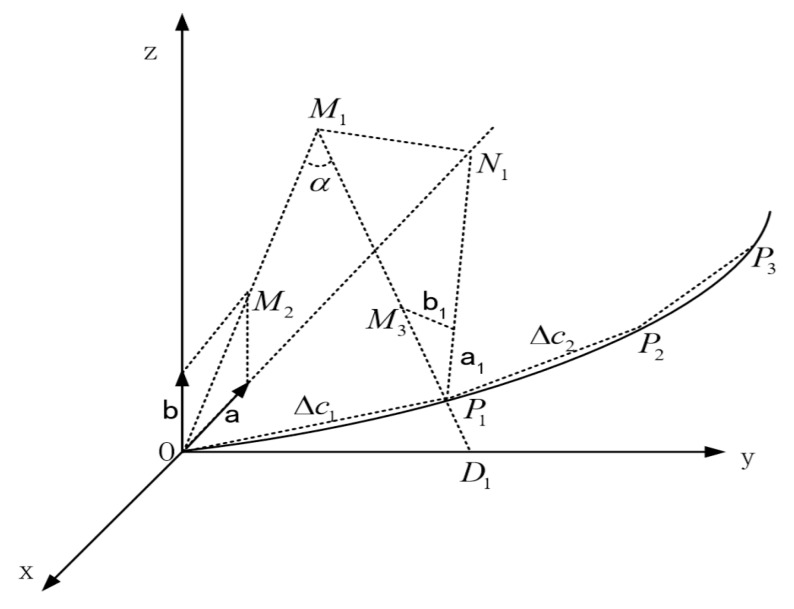
Schematic diagram of space curve fitting.

Put the starting point of the curve at the origin of the coordinate system and fix the direction of the curvature. The space curve function will be deduced by recurrence, as shown in Figure 9. In this case, the tangent line of the first arc is parallel with y-axis. First, measure the curvature in the yoz and xoy planes, which are represented by $\vec{a}$ and $\vec{b}$ and parallel with x-axis and y-axis respectively. *P*_1_ and *P*_2_ are the discrete points. $\Delta c_{1}$ is the length of $\overset{⌢}{OP_{1}}$. When $\lim\Delta c_{1}\to 0$, $\overset{⌢}{OP_{1}}$ can be replaced by straight line *OP*_1_. The space curve of the needle can be worked out by getting the sum of $\vec{OP_{1}}$ and $\vec{P_{1}P_{2}}$.

Take the calculation process of $\vec{OP_{1}}$ as an example. $\vec{OM_{2}}$ is the sum of $\vec{a}$ and $\vec{b}$, which is also the curvature at point *O*. According to the relations between curvature and curvature radius, the space vector $\vec{OM_{1}}$ of the curvature radius at point *O* can be obtained by:

(9) $$\vec{OM_{1}}=\frac{\vec{OM_{2}}}{\left|\vec{OM_{2}}\right|\left|\vec{OM_{2}}\right|}$$

Connect *M*_1_ and *P*_1_ and extend the line until it intersects with y-axis at point *D*_1_. $\vec{OD_{1}}$ is vertical to $\vec{OM_{1}}$. *P*_1_ is the discrete point which means the length of arc $\overset{⌢}{OP_{1}}$ is known. Therefore, ∠α can be obtained by:

(10) $$\alpha =\frac{\Delta C_{1}}{OM_{1}}$$

Since *OD*_1_ is vertical to plane xoz, it is also vertical to $\vec{a}$ and $\vec{b}$, so vector $\vec{OD_{1}}$ can be calculated and also $\vec{M_{1}D_{1}}$.

(11) $$\vec{OD_{1}}=\frac{\vec{a}\times \vec{b}}{\left|\vec{a}\right|\left|\vec{b}\right|}\times \left|OM_{1}\right|\times tan\left(a\right)$$

(12) $$\vec{M_{1}D_{1}}=\vec{OD_{1}}-\vec{OM_{1}}$$

Then $\vec{M_{1}P_{1}}$ and $\vec{OP_{1}}$ can be obtained:

(13) $$\vec{M_{1}P_{1}}=\frac{\vec{M_{1}D_{1}}}{\vec{\left|M_{1}D_{1}\right|}}-\left|\vec{OM_{1}}\right|$$

(14) $$\vec{OP_{1}}=\vec{OM_{1}}+\vec{M_{1}P_{1}}$$

As shown in Figure 9, draw a line $\vec{M_{1}N_{1}}$ to make it vertical to plane *OM*_1_*D*_1_. *M*_1_*N*_1_ intersects with x-axis at point *N*_1_. Next is to figure out vector $\vec{a_{1}}$ and $\vec{b_{1}}$ at point *P*_1_ which are corresponding to $\vec{a}$ and $\vec{b}$. $\vec{a_{1}}$ and $\vec{b_{1}}$ can be obtained by rotating $\vec{a}$ and $\vec{b}$ around *M*_1_*N*_1_ by α. The curvature vector $\vec{a_{2}}$ and $\vec{b_{2}}$ can be calculated according to the curvature at point *P*_1_.

It can be known from Figure 9 that $\Delta M_{1}P_{1}N_{1}≅\Delta M_{1}ON_{1}$. First calculate $\vec{ON_{1}}$, $\vec{M_{1}N_{1}}$, and $\vec{N_{1}P_{1}}$, and then $\vec{a_{1}}$ can be obtained:

(15) $$\vec{ON_{1}}=\frac{\vec{a}}{\left|\vec{a}\right|}\times \frac{\left|\vec{OM_{1}}\right|}{\sin(\tan\frac{\left|\vec{b}\right|}{\left|\vec{a}\right|})}$$

(16) $$\vec{M_{1}N_{1}}=\vec{ON_{1}}-\vec{OM_{1}}$$

(17) $$\vec{N_{1}P_{1}}=\vec{M_{1}P_{1}}-\vec{M_{1}N_{1}}$$

(18) $$\vec{a_{1}}=-\frac{\vec{N_{1}P_{1}}}{\left|\vec{N_{1}P_{1}}\right|}\times \left|\vec{a}\right|$$

Then $\vec{b_{1}}$ can be obtained according to the relations between $\vec{a_{1}}$, $\vec{b_{1}}$ and $\vec{a}$, $\vec{b}$. Assume the norm of the curvature vectors corresponding to $\vec{a_{1}}$ and $\vec{b_{1}}$ is $\left|\vec{a_{2}}\right|$ and $\left|\vec{b_{2}}\right|$, then the curvature vectors $\vec{a_{2}}$ and $\vec{b_{2}}$ can be obtained:

(19) $$\vec{M_{3}P_{1}}=-\left|\vec{OM_{2}}\right|\times \frac{\vec{M_{1}P_{1}}}{\left|\vec{M_{1}P_{1}}\right|}$$

(20) $$\vec{b_{1}}=\vec{M_{3}P_{1}}+\vec{a_{1}}$$

(21) $$\vec{a_{2}}=\left|\vec{a_{2}}\right|\times \frac{\vec{a_{1}}}{\left|\vec{a_{1}}\right|}$$

(22) $$\vec{b_{2}}=\left|\vec{b_{2}}\right|\times \frac{\vec{b_{1}}}{\left|\vec{b_{1}}\right|}$$

The solving procedure of $\vec{P_{1}P_{2}}$ is the same as that of $\vec{OP_{1}}$. Accordingly, $\vec{P_{2}P_{3}}$, $\vec{P_{3}P_{4}}$…can also be obtained. Connecting the vectors is the fitting broken line of the curve. As long as $\lim\Delta c_{1}\to 0$, the fitted line will be accurate.

To solve the curve function of the needle, first is to calculate the curvature radius from the voltage of the gauge at each discrete point. The length of each arc is the length between gauges, which is already known when gluing the gauges. The function of the needle curve then can be deduced according to the equations listed before.

### 3.3. Error Analysis of Curve Fitting

The curve fitting is achieved using multiple arcs to approximate a curve. Since the value of the curvature radius is a continuous variable, this method must have errors. In the section, the layout of the sensors (i.e., how to arrange the discrete points) to keep the error within a certain range is discussed.

#### 3.3.1. The Position Relations between Circles of Curvature and the Curve

Assuming *f*(*x*) is the curve function with a continuous second order derivative, and *f*″(*x_0_*) is not zero. The curvature of the curve is:

(23) $$h(x)=k{\left(x\right)}^{2}=\frac{f^{\prime \prime }{\left(x\right)}^{2}}{{\left(1+f^{\prime }{\left(x\right)}^{2}\right)}^{3}}$$

From (23), it is known that *h*(*x*) and *k*(*x*) have the same poles. According to the values of *h′*(*x*_0_) and *h*″(*x*_0_), there are three conditions:

1. *h′*(*x*_0_) = 0 and *h*″(*x*_0_) > 0, then *k*(*x*) has a minimum at *x*_0_. There must exist a number ε, and *f*(*x*) is outside of the curvature circle of point *x*_0_ in the open interval (*x*_0_ − ε, *x*_0_ + ε).
2. *h′*(*x*_0_) = 0 and *h*″(*x*_0_) < 0, then *k*(*x*) has a maximum at *x*_0_. There must exist a number ε, and *f*(*x*) is inside of the curvature circle of point *x*_0_ in the open interval (*x*_0_ − ε, *x*_0_ + ε).
3. *h′*(*x*_0_) ≠ 0, then *k*(*x*) has no extremum at *x*_0_. There must exist a number ε, and *f*(*x*) is partially outside and partially inside of the curvature circle of point *x*_0_ in the open interval (*x*_0_ − ε, *x*_0_ + ε).

**Figure 10:**
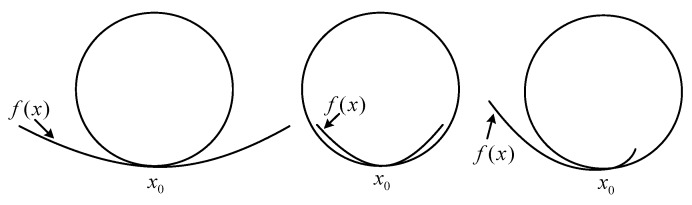
The position relations between a circle of curvature and the curve.

Figure 10 shows the three cases discussed above. If *f^’^′*(*x*) is zero, the curvature at this point is zero. It means it is the demarcation point of a concave-convex curve or on a straight line, which doesn’t change the three conditions.

#### 3.3.2. Error Measurement

When fitting the actual curve *f*(*x*), the first step is to analyze the curvature and work out all the extreme points and their coordinates. Divide the curve at the extreme points and fit every small segment. This section will investigate the fitting error between two extreme points.

**Figure 11:**
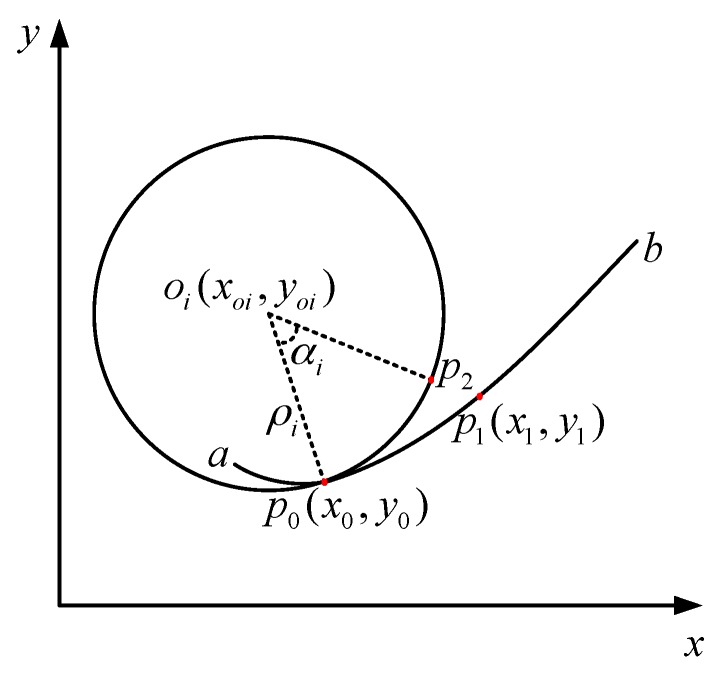
Schematic diagram of error measurement.

There are two conditions in the fitting: starting from point *x*_0_, with *x* increasing, the curve goes inside or outside of the curvature circle. The rest of this part will only take the outside condition as an example, as shown in Figure 11. 

$\overset{⌢}{\mathrm{ab}}$ is a smooth continuous curve and part of *f*(*x*). *a* and *b* are two adjacent curvature extreme points, and the curvature from *a* to *b* is diminishing. Choose a random point *p*_0_ on $\overset{⌢}{\mathrm{ab}}$, and draw a circle where *p*_0_ is the point of tangency. Its center is *o_i_* and its radius is *ρ*_i_. Then, choose two points *p*_1_ and *p*_2_ on $\overset{⌢}{\mathrm{ab}}$ and the circle respectively, making $\overset{⌢}{p_{0}p_{1}}$ and $\overset{⌢}{p_{0}p_{2}}$ have the same length. According to the method proposed in Section 3.3.1, $\overset{⌢}{p_{0}p_{1}}$ is replaced by $\overset{⌢}{p_{0}p_{2}}$ during the fitting. To make sure the error of the curve within a certain range, the error of each segment should be kept under some value. The error of each segment is defined as follow:

(24) $$e_{i}=\sqrt{{\left(x_{1i}-x_{2i}\right)}^{2}+{\left(y_{1i}-y_{2i}\right)}^{2}}$$

where $\left(x_{1i},y_{1i}\right)$ and $\left(x_{2i},y_{2i}\right)$ are the coordinate of $p_{1}$ and $p_{2}$ respectively. According to the fitting method, the actual curve $\overset{⌢}{p_{0}p_{1}}$ is replaced by the curve $\overset{⌢}{p_{0}p_{2}}$. To restrict the error between the actual curve and the fitting one, the error is defined as the distance between point *p*_1_ and *p*_2_. When the tolerant error value is given, point *p*_1_ can be obtained. Assuming the number of segments is *n*, then the total error can be defined as

(25) $$E=\sum_{i=1}^{n}e_{i}=\sum_{i=1}^{n}\sqrt{{\left(x_{1i}-x_{2i}\right)}^{2}+{\left(y_{1i}-y_{2i}\right)}^{2}}$$

The maximum error should be given according to the actual requirement to get an accurate fitting curve.

#### 3.3.3. Curvature Interpolation

In ideal circumstances, adding more discrete points on the needle can minimize the fitting error, which means mounting more sensors. However, too many sensing units means complicated and high-cost system, so the design of the needle should keep a balance between accuracy and cost. 

One possible way to reduce fitting error without increasing sensors is to interpolate between two discrete points to get all points’ curvature values. By using the calculated values in the fitting process, it is easier to get accurate curve. The error of the fitting curve has two affecting factors: the number of sensors and the accuracy of the interpolation method. The number of sensors can be calculated by (25). The rest of this part will discuss the interpolation method. The length of the arc is the independent variable and the curvature is the dependent variable. The needle is smooth and continuous, so it must be guaranteed that the fitting curve is smooth and continuous. That the curvature is continuous means that the curve’s second order derivative is continuous. Polynomial interpolation (over quadratic) can get continuous and smooth curvature value, and can also guarantee the accuracy, so this paper uses the quadratic polynomial interpolation. Using *s* to represent arc length and *k*(s) the curvature. Find three points on the curve segment (*s*_1_, *k*(s_1_)), (*s*_2_, *k*(s_2_)), (*s*_3_, *k*(s_3_)). Taking the three points to Equation (26) and $a_{0},a_{1},a_{2}$ can be calculated.

(26) $$k(s)=a_{0}+a_{1}\times s+a_{2}\times s^{2}$$

## 4. Experiment and Results

This section experiments on the real-time detection of a bevel flexible needle’s curve shape. First is to calibrate the strain gauges. Then calculate the curvature according to the voltage. Finally, get the curve shape by curve fitting with the curvature of discrete points. It is known that it is difficult to detect the shape of a thin metal needle. Therefore, a methodology of comparing with standard curves is proposed in the planar case study (Section 4.2). For the experiments in artificial tissue (Section 4.3), a visual system is employed to describe the trajectory of the needle, in which the extracted visual curve is compared with the fitting curve result based on the gauge sensing information. For the experiments of spatial curve, since it is extremely difficult to reconstruct, only qualitative experiments were performed to validate the feasibility of the proposed methodology. 

### 4.1. Calibration of the Strain Gauge

**Figure 12:**
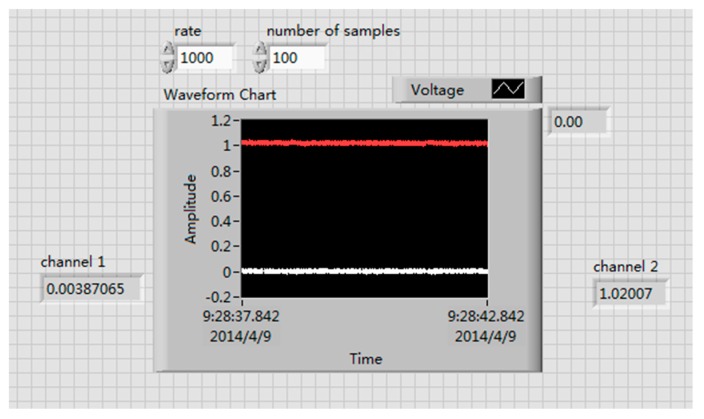
LabVIEW program.

The relations between voltage and curvature is U = K∙*k*. In the experiment, the resistance values were not exactly the same, and there was no bridge balancing section in the circuit, so the initial voltage was not zero. The corrected equation is U = U_0_ + K∙*k*. This paper made a LabVIEW program to collect and record voltages, as shown in Figure 12.

**Figure 13:**
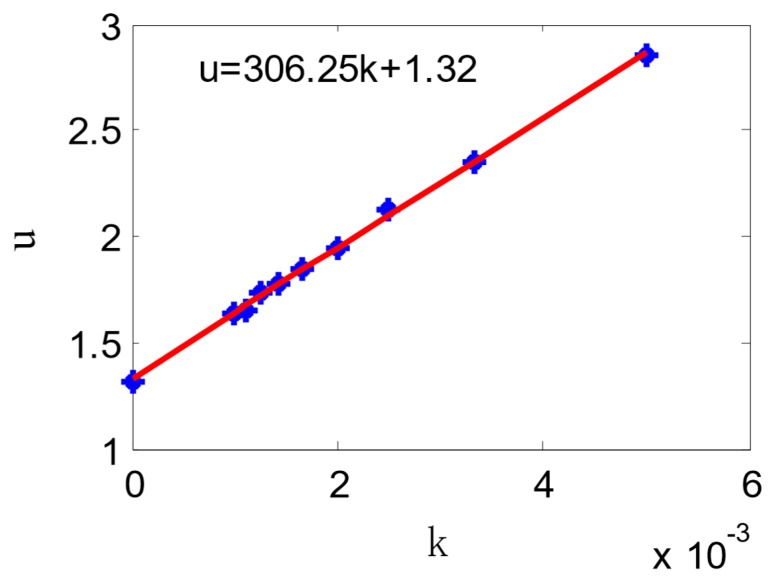
Voltage-curvature relation curve.

The radius is *ρ* = ∞, 1000 mm, 900 mm, 800 mm, 700 mm, 600 mm, 500 mm, 400 mm, 300 mm, and 200 mm. Figure 13 shows different curvatures and their voltages. After fitting the points with the least square method, K and U_0_ was calculated as 258.7568 v∙mm, and 0.9749 v respectively.

### 4.2. Single Arc Experiment

**Figure 14:**
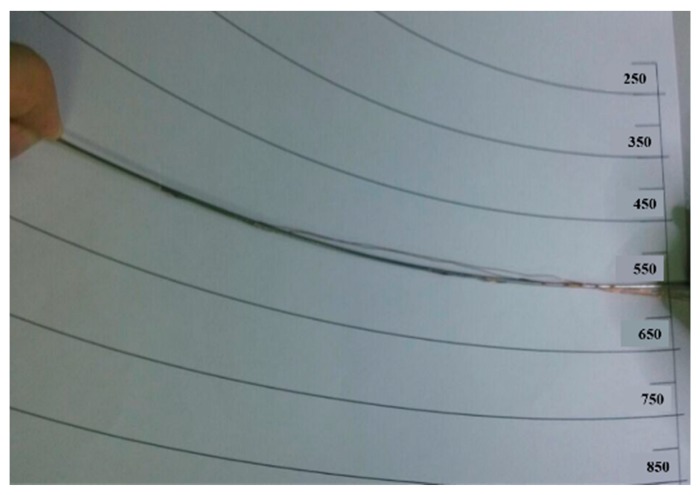
Single arc experiment.

**Figure 15:**
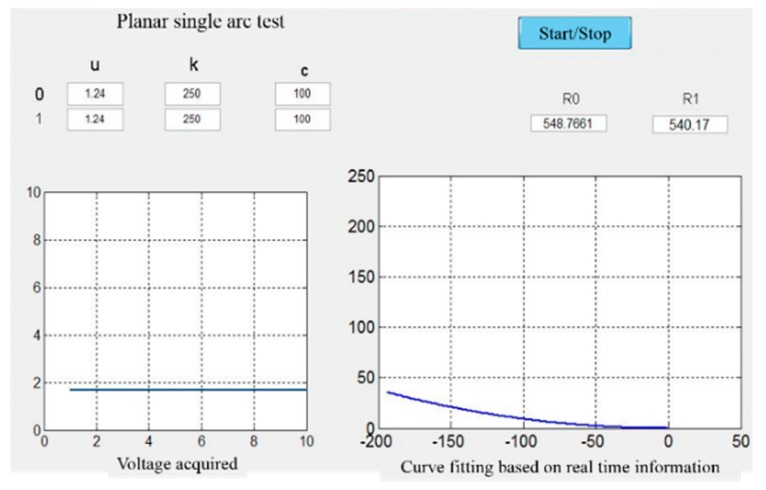
Experiment results.

The simplest case that the needle curve was a single arc was validated first. Theoretically, since the length of the needle is given, the curve shape can be easily got with only one strain gauge. However, in this experiment, the bending of the needle was performed by human, i.e., the shape was not an ideal arc. Therefore, two gauges were glued to the needle. The needle was 200 mm in length, and the two gauges were glued at 50 mm and 150 mm points separately. Bend the needle into an arc as shown in Figure 14, whose radius was 550 mm. Figure 15 is the needle figure on computer, and the two radiuses were 548.7661 mm and 540.17 mm respectively, which are very close to the real value.

### 4.3. Experiment of Needles in Tissue

**Figure 16:**
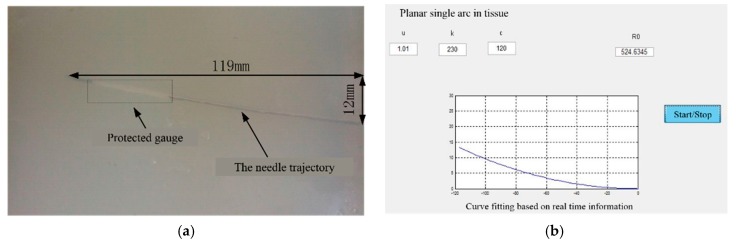
(**a**) Experiment of needle in tissue; (**b**) experiment of needle in the computer image.

In this section, the detection of a needle in artificial tissue was evaluated. The artificial tissue was wet inside, which might cause negative effects to stain gauges. Therefore, the strain gauges were covered with Teflon tape to protect them from damage during the experiment. By using the experiment setup shown in Figure 1, the flexible needle was inserted into the artificial tissue via the linear motion actuated by the lead-screw. The needle was inserted into the tissue by 120 mm, which was controlled by the main control computer. Figure 16a shows the needle in the tissue, which is a semitransparent material made of agar powder. The gauge glued on the needle body sensed the deflection signal, which was the feedback to the main computer. Figure 16b shows the image worked out by computer. The results showed that the curve fitted by the computer didn’t coincide with the real curve completely. The reasons why there was an error could be: First, the artificial tissue had a nonnegligible effect on the gauges, making the collected signals instable. Second, the needle was not in a plane, but rather a space curve, which was hard to see with naked eyes. The deviation of the needle point from the plane was less than 2 mm, so it was acceptable.

### 4.4. Space Curve Experiment

**Figure 17:**
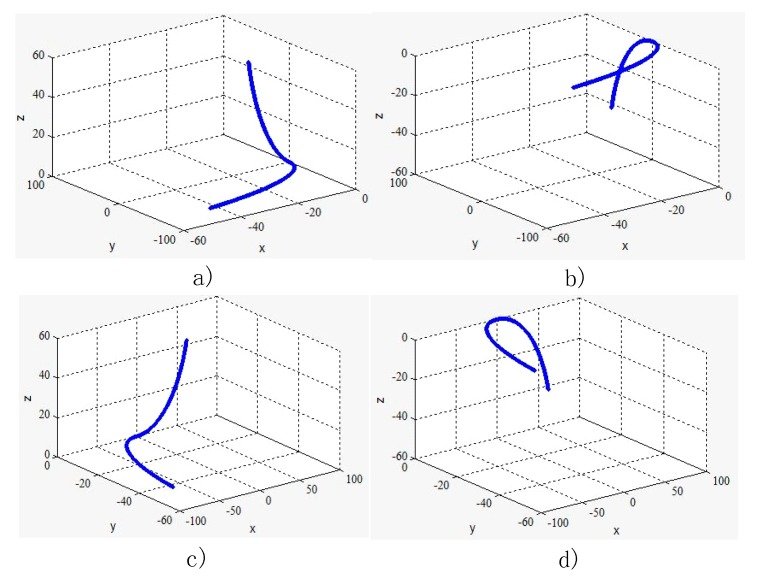
Double arc curves.

In this section, the detection of a double-arc needle was evaluated considering the difficulty of make the curve by human hand. The space curve can be divided in to four groups as shown in Figure 17.

**Figure 18:**
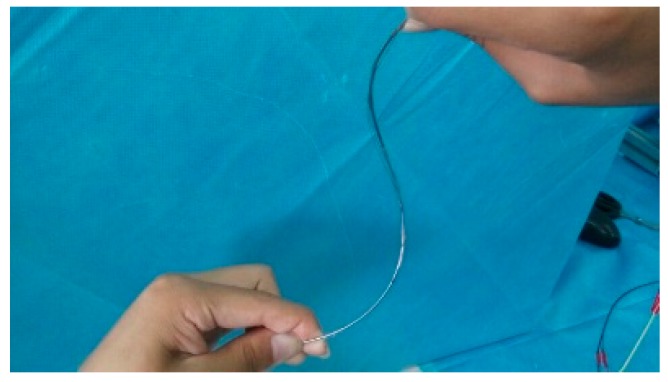
Double arc experiment.

**Figure 19:**
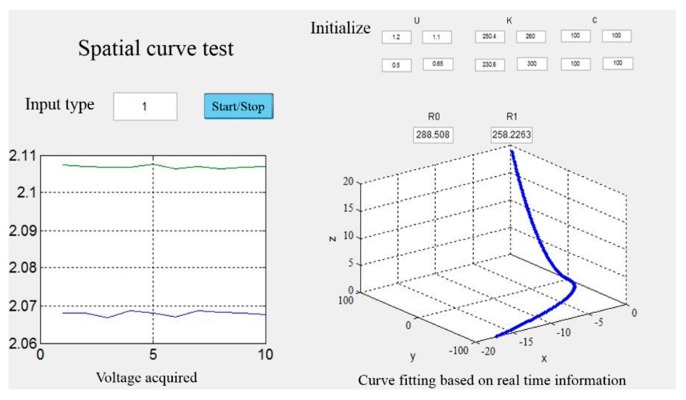
Experiment results.

In all these four situations, the planes of each arc are vertical to each other. This paper only examined the first case in Figure 17a. Figure 18 is the experimental picture and Figure 19 is the computer image.

## 5. Conclusions

To achieve the flexible needle real-time detection without medical imaging equipment, the strain gauges is first integrated in the flexible needle puncture system to realize feedback control during the puncture surgeries. The whole system integrated the mechanism, actuation, sensing, and control into a completed system. This paper researched on the recognition of the needle by curve fitting based on the curvature values which were collected by strain gauges. The feasibility of this thought is verified by the experiment. The curve fitting technique based on curvature has errors and is limited to arc like curves. Future work can focus on advanced curve recognition method to apply for more complicated situations.

## Figures and Tables

**Table 1:** Parameters of the flexible needle.

Parameter	Symbol	Value
Young’s modulus	E	50 Gpa
Diameter	D	1.2 mm
Bevel	α	15°
Length	L	120 mm
